# A blinded clinical study using a subepidermal moisture biocapacitance measurement device for early detection of pressure injuries

**DOI:** 10.1111/wrr.12790

**Published:** 2020-01-21

**Authors:** Henry Okonkwo, Ruth Bryant, Jeanette Milne, Donna Molyneaux, Julie Sanders, Glen Cunningham, Sharon Brangman, William Eardley, Garrett K. Chan, Barbara Mayer, Mary Waldo, Barbara Ju

**Affiliations:** ^1^ Seacliff Healthcare Center Los Angeles California; ^2^ Grand Park Convalescent Hospital Los Angeles California; ^3^ Vermont Convalescent Care Center Los Angeles California; ^4^ Providence Health Care Spokane Washington; ^5^ Tissue Viability & Community Research Service, Nursery Park Health Centre Northumbria NHS Trust, Northumberland UK; ^6^ Thomas Jefferson University Hospital Philadelphia Pennsylvania; ^7^ Gwynedd Mercy University Gwynedd Valley Pennsylvania; ^8^ St Bartholomew's Hospital, Barts Health NHS Trust London UK; ^9^ Good Samaritan Regional Medical Center Corvallis Oregon; ^10^ SUNY Upstate Medical University and Loretto Health and Rehabilitation Syracuse New York; ^11^ Department of Trauma and Orthopaedics Middlesbrough James Cook University Hospital Middlesbrough UK; ^12^ Stanford Health Care Stanford California; ^13^ Providence Portland Medical Center Portland Oregon

## Abstract

This study aimed to evaluate the sensitivity and specificity of subepidermal moisture (SEM), a biomarker employed for early detection of pressure injuries (PI), compared to the “Gold Standard” of clinical skin and tissue assessment (STA), and to characterize the timing of SEM changes relative to the diagnosis of a PI. This blinded, longitudinal, prospective clinical study enrolled 189 patients (n = 182 in intent‐to‐treat [ITT]) at acute and post‐acute sites (9 USA, 3 UK). Data were collected from patients' heels and sacrums using a biocapacitance measurement device beginning at admission and continuing for a minimum of 6 days to: (a) the patient developing a PI, (b) discharge from care, or (c) a maximum of 21 days. Standard of care clinical interventions prevailed, uninterrupted. Principal investigators oversaw the study at each site. Blinded Generalists gathered SEM data, and blinded Specialists diagnosed the presence or absence of PIs. Of the ITT population, 26.4% developed a PI during the study; 66.7% classified as Stage 1 injuries, 23% deep tissue injuries, the remaining being Stage 2 or Unstageable. Sensitivity was 87.5% (95% CI: 74.8%‐95.3%) and specificity was 32.9% (95% CI: 28.3%‐37.8%). Area under the receiver operating characteristic curve (AUC) was 0.6713 (95% CI 0.5969‐0.7457, *P* < .001). SEM changes were observed 4.7 (± 2.4 days) earlier than diagnosis of a PI via STA alone. Latency between the SEM biomarker and later onset of a PI, in combination with standard of care interventions administered to at‐risk patients, may have confounded specificity. Aggregate SEM sensitivity and specificity and 67.13% AUC exceeded that of clinical judgment alone. While acknowledging specificity limitations, these data suggest that SEM biocapacitance measures can complement STAs, facilitate earlier identification of the risk of specific anatomies developing PIs, and inform earlier anatomy‐specific intervention decisions than STAs alone. Future work should include cost‐consequence analyses of SEM informed interventions.

## 1 INTRODUCTION

Pressure injuries (PI) are a widespread and serious complication of reduced patient mobility. Annually, PIs occur in more than 2.5 million US patients, of whom approximately 60  000 die due to infection and other sequelae.^1^ Due to the substantial impacts of PIs on patient quality of life, recovery, and lengths of stay, PI prevention is prioritized by providers and policy makers.^2^, ^3^, ^4^, ^5^ The United States' Agency for Healthcare Research and Quality' (AHRQ) statistics, however, show PIs being the only Hospital Acquired Condition whose incidence worsened during 2014‐2017.^6^ The overall costs of PIs in the United States are estimated to exceed $26.8 billion,^7^, ^8^, ^9^ with per‐patient costs ranging from $500 to $70 000.^10^

A combination of PI risk assessments, supplemented by skin and tissue assessments (STAs) and mechanical offloading (such as patient repositioning and the use of pressure redistributing mattresses and medical devices) comprises most PI prevention programs.^1^ The use, benefits, and limitations of risk assessment tools (RATs) are well documented elsewhere^11^, ^12^, ^13^, ^14^, ^15^ and other than their use in screening patients for initial risk, are not the subject of this manuscript. STA appraises skin color, blanchability, temperature, hardness, and other visible or palpable indicators of injury. The international pressure ulcer advisory panel's—US National Pressure Ulcer Advisory Panel/European Pressure Ulcer Advisory Panel/Pan Pacific Pressure Injury Alliance (NPUAP/EPUAP/PPPIA)—global 2014 Clinical Practice Guideline, states, “The condition of skin and underlying tissue can serve as an indicator of early signs of pressure damage, therefore routine STA provide an opportunity for early identification and treatment of skin alterations, especially pressure ulcers.”^11^ Clinical judgment of nurses, informed by risk tools and STA, however, “achieved inadequate capacity to assess PU risk”^12^ and suffered from “high inter‐examiner variability.”^12^, ^13^ Clinical judgment has a sensitivity of 50.6% and specificity of 60.1%.^13^ Because of the skill dependency of STA, correct identification of a stage I pressure ulcer has been observed as low as 60% in a diverse group of 1452 nurses.^14^ Latency of visual and palpable signs do not address nonvisible cues of tissue damage: PI often occur without prior visual and palpable cues appearing in time to prevent them. In patients at high risk for pressure ulcer formation, nonblanchable erythema can develop in as little as 2 hours from injury.^15^ Due to the subjective, latent nature of STAs, there is a clear need for an objective, point‐of‐care tool for diagnosing or assessing developing PIs on at‐risk patients.

A change in subepidermal moisture (SEM) due to local edema or accumulation of interstitial fluid is a biomarker of a developing PI and precedes the appearance of visible or palpable skin changes by approximately 3‐10 days.^16^, ^17^, ^18^, ^19^, ^20^, ^21^ Electrical biocapacitance of human tissue varies with interstitial moisture content; therefore, SEM changes manifest as changes in biocapacitance, which can be measured by point‐of‐care devices.^19^, ^22^, ^23^ A biocapacitance measurement device^24^ that has previously shown inter‐operator and inter‐device reliability in detection of SEM changes (*R* ≥ 0.8), as well as agreement with ultrasound detection of hypoechoic lesions consistent with PIs, supporting the potential utility of this device.^19^, ^21^, ^25^ Independent studies suggest that a biocapacitance measurement is a useful adjunct to clinical STAs, particularly for prediction of developing PIs, and the data, when used to initiate anatomy‐specific interventions may reduce the incidence of hospital‐acquired PI.^18^, ^20^, ^26^, ^27^, ^28^, ^29^ The comparative sensitivity and specificity of biocapacitance measurements in detecting PIs relative to the current reference standard of clinical STA is however, not well characterized. This longitudinal clinical study was conducted to evaluate the sensitivity and specificity of assessing SEM using a biocapacitance measurement device in accordance with regulatory guidelines for new diagnostic tests,^30^ and further, to characterize the timing of SEM changes compared to the diagnosis of PI by STA scales.

## 2 MATERIALS AND METHODS

### 2.1 Study design

This multisite, blinded, prospective, longitudinal clinical study was conducted at 12 inpatient facilities (six acute care and three post‐acute care settings in the United States [77.8%; n = 147] and three acute care settings in the United Kingdom [22.2%; n = 42]) consistent with good clinical practices standards set by the USA's Code of Federal Regulations (21 CFR 812—*Investigational Device Exemption*; United States 21 CFR 50—*Protection of Human Subjects*; United States 21 CFR 56—*Institutional Review Boards*), International Standards (ISO 14155:2011*—Clinical Investigation of Medical Devices for Human Subjects—Good Clinical Practice*); and, the International Conference on Harmonization of Good Clinical Practice Guidelines and the Declaration of Helsinki.

Subjects were tracked from enrollment with no observed PI to discharge. Discharge was precipitated by any of three events: (a) confirmation of a pressure injury by an expert “Specialist”; (b) discharge from the facility after a minimum of 6 days of observation; or (c) satisfying the maximum enrollment of 21 days. The prospective design tested two objectives; the first, that measured changes in the SEM biomarker are associated with the later manifestation of a PI (stage 1, stage 2, deep tissue injury) to 70% sensitivity and 55% specificity with 95% confidence compared to the reference standard of clinical STA. Second, that the SEM biomarker gives notification of such changes prior to an expert skin assessed PI manifesting at the skin's surface.

A subtle distinction of consequence is that the first objective was based on the detection of a biomarker (SEM) of the later onset of a stageable PI. No designation of a PI preceding stage 1 in the etiological process exists in any published guidelines: Stage 1 being the first available stage of PI useable to establish sensitivity and specificity.

Protocol design was informed by an independent investigator study^20^ which pointed toward viability of clinical interpretation using the threshold cutoff value of the biocapacitance device (device) used in this study (Δ ≥0.6, explained below). O'Brien's study demonstrated that the device detects changes in pressure injured skin and tissue earlier than nurses' STA alone. Of the 47 patients enrolled in O'Brien's study, 40.43% (n = 19) study participants with consecutive days of elevated SEM levels (defined in O'Brien's study as, “a difference of greater than 0.5 between the lowest and highest [SEM] values recorded”. Although using different descriptions of the delta calculation, the definitions of the SEM delta as >0.5 or Δ ≥0.6 are mathematically equivalent), went on to develop signs of a pressure injury. Further, the use of the device identified changes to the SEM biomarker on average 3.9 days earlier than the nurses' STAs confirming a PI.^20^

Study participants provided informed consent in writing. Three categories of clinical staff were organized to execute the trial at each site. Each site's principal investigator, or their formally appointed designee, oversaw the conduct of the entire trial for their site. “Specialists,” comprising experienced wound care specialists with accreditation in wound and ostomy or tissue viability care performed risk, skin, and PI diagnoses. Diagnoses by Specialists formed the “gold standard” of the existence or absence of a PI. It was against these gold standard diagnoses that device readings were evaluated. SEM biocapacitance measurements were performed by a separate group of, “Generalists” who received training in operation of the device by the device manufacturer and were tested for proficiency prior to initiation of the study. Generalists were clinical practitioners, generally Registered Nurses, Surgical Assistants, and Medical Doctors and were prohibited for the purposes of the trial from participating in wound care related clinical decisions for enrolled patients. Specialists and Generalists' data were blinded from each other. Each site's protocol compliance and data integrity were overseen and audited by a, “Study Coordinator.” All data were loaded to Medrio, an electronic data management system (Medrio Inc., California, USA), and locked; consistent with good clinical practices. A full post hoc audit of all data points was conducted by a Regulatory Body; four minor audit findings were noted relating to two sites, all of which were immediately able to be corrected. No findings affected the integrity of the data. No audit findings were noted for the Sponsor.

Withholding “standard of care” prevention or treatment interventions from enrolled subjects would have been unethical. Interventions were applied to all patients consistent with facility Standard of Care protocols. No interventions were permitted to be withheld nor were any interventions triggered by device readings. Data about such interventions were collected at every visit to allow assessment of potentially confounding effects.

Etiological studies show that early pressure damage does not always manifest into a visible PI.^19^, ^31^, ^32^, ^33^ Researchers of early stage PIs and PI biomechanics demonstrated the inherent reversible nature of early pressure damage.^19^, ^31^, ^32^, ^33^ Reversibility and self‐resolution are known phenomena PI.^19^, ^31^, ^32^, ^33^ Some early damage will progress to a PI and some will reverse back to a healthy state, depending on a variety of factors including a patient's overall health and whether an intervention is taken to alleviate pressure and or shear. Moreover, some pressure damage can be (a) stable, (b) not progressing, or (c) reversing.^31^ Ultimately this means that increases in SEM delta values will not always lead to a PU, but a PI will be preceded by a change in SEM. Complications to end‐point analysis from these etiological realities were considered during trial design and in interpretation of the data.

Eligible study participants were patients ≥55 years of age who could be followed for at least six consecutive days and were determined to be at risk of developing PIs based on a score from validated risk assessment scales (Braden scale, < 15; Waterlow scale, ≥ 10; or Norton scale ≤18), poor nutrition, poor mobility (chair or bed‐bound), or completion of a recent medical procedure requiring subsequent immobilization (eg, surgery). The minimum follow‐up period of 6 days was chosen to provide a sufficient window of time for PI development, which evolves over approximately 1‐5 days.^34^, ^35^

Patients were excluded from study participation for any of the following: existing PIs; broken skin at either the sacrum or heels; moisture lesions or incontinence‐associated dermatitis (IAD); and biomechanical or other limitations preventing protocol‐driven assessments. Use of the device over broken skin was contraindicated, hence, in part, the exclusion of IAD.

The study focused entirely on heels and sacrum; anatomies accounting for more than 50% and up to 87% of reported PIs.^23^, ^24^, ^36^, ^37^

### 2.2 Study procedures

Participants were followed for at least 6 and up to 21 days unless a pressure injury occurred in the intervening period, in which case they were discharged per protocol. No patients were followed after developing a PI in the study. Each day, participants were assessed for risk of a PI by Specialists using one of three established scales, depending on facilities' existing standard of care: the Braden Scale for Predicting Pressure Sore Risk,^38^ the Waterlow Pressure Ulcer Prevention/Treatment Policy score,^39^ or the Norton Pressure Sore Risk‐Assessment Scale Scoring System.^40^ Skin temperature, erythema, edema, consistency (induration/hardness) in relation to surrounding tissue moisture, turgor, and health, as well as patient‐reported pain, were assessed in accordance with established guidelines.^1^, ^41^ Incidents of PIs were staged as described elsewhere.^1^

SEM biocapacitance measurements were obtained from the sacrum **(**Figure S1) and heels (Figure S2 **)**. Results reflective of the underlying distribution of moisture depend on multiple readings from areas over and immediately contiguous to the anatomy. Six readings were therefore obtained from the sacral area and at least three readings were obtained from each heel.^42^

### 2.3 Device

The principles underlying the use of biocapacitance as an indirect measure of localized SEM and edema have been reviewed previously.^19^, ^43^ Biocapacitance varies with interstitial fluid content. Measurements of tissue biocapacitance may be used as an indicator of SEM.^24^ The SEM Scanner (device) (Figure S3 **)** (Bruin Biometrics, LLC, Los Angeles, CA, USA) technology assesses the fluid contents of epidermis and subdermal tissues. The device makes a direct steady‐state measurement of the capacitance of its sensor, which is affected by the equivalent dielectric constant of the material (ie, the layered tissue structures) that is within the electric field between the sensor electrodes to a depth of 0.15 in. (4 mm) and converts the biocapacitance from SI units to a score.^24^ Two values are displayed on the device's screen, an individual value for each single scan, and after three readings are taken, the SEM Delta (ΔSEM), calculated as the difference of the minimum and maximum SEM values obtained at and immediately contiguous to an anatomical site. Calculation of a “delta” value compares measurements from several sites, some of which will be healthy tissue, compensates for systemic changes, overcomes the limitation of inter‐ and intra‐patient variability and provides a measure of tissue health condition.^24^ *

The range of device values is 0.2 to 4.0, with known tolerance of ±0.2. Prior sponsor studies determined a threshold delta value: Δ < 0.6 indicating lower risk for a pressure injury at the anatomy and Δ ≥ 0.6 indicative of increased risk for pressure ulcers at the anatomy being measured.

### 2.4 Statistical analysis

This study was powered to detect at least 70% sensitivity and 55% specificity of the device compared to the reference standard of clinical STA, with 95% confidence. A total of 189 patients were enrolled, of which 96.3% (n = 182) patients were listed as intent‐to‐treat (ITT) Figure S4. Within the 12 sites included in the study, the trials were completed in sites of service shown in Table 1.

**Table 1:** Sites of service for intent‐to‐treat population

Site of service	Subjects (n = 182) and % of total in the ITT
Orthopaedic trauma	n = 26 (14%)
Medical surgery	n = 50 (27%)
Long‐term care	n = 58 (32%)
Intensive care units (ICU)	n = 17 (9%)
Rehabilitation	n = 7 (4%)
Neurologic care	n = 15 (8%)
Other/mixed	n = 9 (5%)

Abbreviations: ITT, intent‐to‐treat.

Sensitivity and specificity analyses were completed on the 182 ITT population and on 170 per protocol (PP) patients. Division of enrolled participants into the ITT and per‐protocol (PP) analyses is detailed in Figure S4. The ITT analysis includes all subjects correctly enrolled regardless of subsequent nonmajor protocol deviations (eg, missed day by Specialist 37.1% and missed day by Generalist 29%). The PP cohort were subjects from the ITT population who met all inclusion and exclusion criteria, did not have protocol violations or deviations (eg, missed days, nonanalyzable data), and had both comparable days of data set by Specialists and Generalists. The PP cohort represented the highest quality data set; with complete data from all three anatomical locations and all daily evaluations completed by Specialists and Generalists for a minimum of 6 days with no gaps.

A Positive Detection was defined as, “Observation of two or more SEM deltas Δ ≥0.6 from three consecutive series of device readings prior to pressure ulcer diagnosis by clinical judgment of the Specialist”, and negative detection was defined as, “Observations of two or more SEM deltas Δ<0.6 from three consecutive series of device readings prior to no pressure ulcer diagnosis by clinical judgment of the Specialist.” For the purposes of endpoint analysis, a “Valid Series” of device measurements was considered when 3 days of device measurements were performed with no more than 1 day missing between these 3 days of measurements. A valid series comprised no more than four observation days of device measurements. The following are examples of valid series under this definition with “X” representing a study day with device measurements and “0” representing a day with no device measurements.

Example A: Three days of measurements without any missing days: X X X.

Example B: Four days of measurements with 1 day missing:

0 X X X

X 0 X X

X X 0 X

X X X 0

Positive and negative results from the Scanner were compared to Specialist assessments of patients' skin and tissue. These results formed the classes required to compute statistics for the first endpoint. Results were classed:

- True positive (TP): a pressure ulcer as confirmed by STA and a localized SEM positive delta of Δ ≥ 0.6
- True negative (TN): no pressure ulcer as confirmed by STA and a localized SEM negative delta Δ < 0.6
- False negative (FN): a pressure ulcer as confirmed by STA and a localized SEM positive delta Δ < 0.6.
- False positive (FP): no pressure ulcer as confirmed by STA and a localized SEM negative delta of Δ ≥ 0.6.

Specificity and sensitivity for both the ITT and PP populations were calculated based on counts of true negative (TN), true positive (TP), false negative (FN), and false positive (FP) findings using the method described by the US Food and Drug Administration,^30^ such that:

$$\text{Sensitivity}=100\times \frac{\mathrm{TP}}{\mathrm{TP}+\mathrm{FN}}$$

and;

$$\text{Specificity}=100\times \frac{\mathrm{TN}}{\mathrm{FP}+\mathrm{TN}}.$$

Two‐sided 95% confidence intervals were calculated using the exact method. Statistical analysis was performed using SAS version 9.4 (SAS Institute Inc., Cary, NC) by an independent biostatistician according to a prospectively defined statistical analysis plan. At the request of the FDA, an additional sensitivity and specificity analysis was conducted using the Bootstrap method. The bootstrap method was applied by sampling, with replacement, from the original data set. Sampling was done on a per subject basis such that all records for a randomly chosen subject were extracted. One thousand data sets were generated using this method, each with the same number of subjects as the original data set.^44^

A *post hoc* receiver operating characteristic (ROC) curve analysis was conducted to augment the description of diagnostic accuracy. ROC curves estimate and report all combinations of sensitivity and specificity the test is able to provide.^45^ Results are expressed as a statistic, the “area under the curve” (AUC), a value ranging from 0 to 1 (ie, 0%‐100%) where 0.5 (ie, 50%) represents randomness. Values above 0.5 trend toward increasing diagnostic certainty for the test.

The measure for secondary endpoint (“time to detection”) was the number of days between pressure ulcer diagnosis by clinical judgment of the Specialist and the first day of measuring a delta (Δ) value ≥0.6.

### 2.5 Limitations of the evaluative rubric of sensitivity and specificity

The classic approach^30^, ^46^ to evaluating the accuracy of a diagnostic accuracy is to compare the results of the test under evaluation (index test) with the results of a reference standard; the best available method to determine the presence or absence of the condition or disease of interest. This reference standard is ideally, a “gold standard”, namely one that is without error. The performance of a new thermometer, for example, can be tested against an existing, objective measurement of temperature. A pure test for a new diagnostic device benefits from assessing a disease state that is not susceptible to being confounded by reversal or healing and can be objectively diagnosed, without error.

The rubrics of “sensitivity” and “specificity” do not neatly apply to the epistemological objectives central to this study, but nonetheless remain the paradigm statistical measures for a new diagnostic. The use of specificity as an end point was recognized, before study inception, as a worst‐case assessment for the SEM test because it classes all results in which a pressure ulcer did not visibly manifest (STA negative) but where changes in SEM were observed (SEM positive) as false positive results. No presently available alternative to the design was possible.

## 3 RESULTS

### 3.1 Study population

One‐hundred and eighty‐nine (n = 189) participants (46.7% males and 53.3% females) were enrolled, primarily from US sites (77.8% US vs 22.2% UK, respectively). Seven participants' data were not analyzable, resulting in an ITT population of 182 (Figure S4). The removal occurred before data analysis, which was performed following the a priori established statistical analysis plan. The reason for removal of six subjects' data was necessary because the delta values for each SEM reading point had been entered erroneously and could not be corrected by study staff or site monitors *post hoc*. One subject with an existing pressure ulcer (exclusion criteria, 1) was also erroneously enrolled into the study for 1 day and therefore did not meet eligibility. This was also a protocol deviation which was noted, reported and rectified by the principal investigator at the site.

With the exception of one site that used the Waterlow Scale, all study sites used the Braden scale for risk assessment as part of routine care. Demographic data are shown in Table 2.

**Table 2:** Participant demographics

	n	% or mean ± SD
Body mass index (kg/m^2^)		
Total	182	26.8 ± 7.65
Male	85	27.4 ± 6.70
Female	97	26.2 ± 8.39
Age (years)		
Total	182	76 ± 11
Male	85	73 ± 11
Female	97	79 ± 11
Sex		
Male	85	46.7%
Female	97	53.3%
Race		
White or Caucasian	121	66.5%
Black/African American	8	4.40%
Asian	44	24.2%
American Indian/Alaskan Native	1	0.55%
Pacific Islander/Native Hawaiian	2	1.10%
Unknown	2	1.10%
Other	4	2.20%
Ethnicity		
Non‐Hispanic/Latino	158	86.8%
Hispanic/Latino	8	4.40%
Unknown	12	6.59%
Does not wish to provide	4	2.20%
Risk assessment scores		
*Braden Scale*		
Total	166	91.2%
Very high risk (≤9)	15	9.04%
High risk^10^, ^11^, ^12^	50	30.1%
Moderate risk^13^, ^14^	71	42.8%
Mild risk^15^, ^16^, ^17^, ^18^	20	12.1%
Not at risk (> 18)	2	1.20%
Missing	8	4.82%
*Waterlow Scale*		
Total	16	8.79%
Very high risk (≥20)	5	31.3%
High risk^15^, ^16^, ^17^, ^18^, ^19^	3	18.8%
At risk^10^, ^11^, ^12^, ^13^, ^14^	6	37.5%
Not at risk (< 10)	2	12.5%

### 3.2 Skin and tissue assessments

STAs were performed on 437 unique anatomical locations (36.6%, n = 160 left heels; 36.4%, n = 159 right heels; and 27%, n = 118 sacral locations) during the course of the study. An interest in determining the existence or absence of differences in SEM readings between heels resulted in the preponderance of readings being at the heels. Additionally, some patients expressed emotional sensitivities to assessment of their sacral areas, which limited the number of sacral assessments. Applying PI staging per published guidelines,^1^ a total of 26% (n = 48/182) of the ITT population were diagnosed with a PI (Table 3), and the incidence by anatomical site was 11% (n = 48/437 (Table 5).

**Table 3:** Classification of pressure injuries diagnosed by STA

	All		Sacrum		Heels	
PI classification	n	%	n	%	n	%
Stage I	32	66.7	12	25	20	41.7
Stage II	3	6.3	3	6.3	0	0.0
Stages III‐IV	0	0.0	0	0.0	0	0.0
Unstageable	2	4.2	0	0.0	2	4.2
Suspected deep tissue injury	11	22.9	1	2.1	10	20.8
Total	48		16		32	

Table 4 provides a baseline skin profile of the enrolled subjects. Over 85% of the ITT population with available data were identified as having normal skin color and, a small percentage showed evidence of redness at the sacrum (12.60%), left heels (6.67%), and right heels (4.88%). Over 99% of the ITT indicated that the skin was blanchable at day 0. Enrollment data also show a majority of subjects were observed as having normal skin temperature, edema, induration, moisture, turgor, and health with no gross observations of bruising, cracked skin, abrasions, dehydration, pain; and moisture lesions were absent at the sacrum.

**Table 4:** Baseline skin profile of enrolled subjects at day 0 (enrollment)

Skin characteristics	ITT (N = 182)		
	Sacrum (n = 127)	Left heel (n = 165)	Right heel (n = 164)
Skin color	n (%)	n (%)	n (%)
Cyanosis	0 (0%)	1 (0.61%)	1 (0.61%)
Darker tone	2 (1.57%)	2 (1.21%)	2 (1.22%)
Normal color	109 (85.83%)	143 (86.67%)	144 (87.80%)
Paleness	3 (2.36%)	11 (6.67%)	11 (6.71%)
Redness	16 (12.60%)	11 (6.67%)	8 (4.88%)
Blanching			
Blanchable	126 (99.21%)	165 (100.0%)	164 (100.0%)
Nonblanchable	1 (0.79%)	0 (0%)	0 (0%)
Skin characteristics			
Skin temperature—normal	116 (91.34%)	143 (86.67%)	144 (87.80%)
No edema	121 (95.28%)	141 (85.45%)	140 (85.37%)
No induration	126 (99.21%)	165 (100.0%)	163 (99.39%)
Skin moisture—normal	113 (88.98%)	134 (81.21%)	134 (81.71%)
Skin turgor—normal	125 (98.43%)	160 (96.97%)	158 (96.34%)
Tissue health—skin intact	125 (98.43%)	164 (99.39%)	164 (100.0%)
No other characteristics	119 (93.70%)	152 (92.12%)	149 (90.85%)
Pain free (score 0)	107 (84.25%)	107 (64.85%)	107 (65.24%)
Moisture lesion—absent	125 (98.43%)	0 (0%)	0 (0%)

*Note*: Percentages for STA data were calculated using number of accessible anatomical locations at the time of assessment as the denominator.

Abbreviations: STA, skin and tissue assessment; ITT, intent‐to‐treat.

### 3.3 SEM results

True positive, True Negative, False Positive, and False Negative classification results for the ITT population are shown in Table 5. In the ITT and PP populations, respectively, sensitivity relative to STAs was 87.5% (95% CI: 74.8%‐95.3%) and 84.2% (95% CI: 68.8%‐94.0%). Calculated specificity was 32.9% (95% CI: 28.3%‐37.8%) in the ITT population and 32.9% (27.0%‐39.3%) in the PP population. Using the Bootstrap method, sensitivity was 87.4% (95% CI: 77.8%‐96.7%) and specificity was 33% (95% CI: 27.6%‐38.7%). Incidence by anatomical site was 48/437 (11%). AUC for the ITT was 67.13% (95% CI: 59.7%‐74.6%). Positive predictive value (PPV) and negative predictive value (NPV) are the proportions of positive or negative results in diagnostic tests that are true positive and true negative results, respectively. For this SEM test, the PPV of SEM tests was 14%, and the NPV was 96%.

**Table 5:** Final True positive, True Negative, False Positive, and False Negative classification results for the ITT population

ITT analysis (n = 182; total anatomical locations [437])						
	All locations		Sacrum		Heel	
	SA+	SA−	SA+	SA−	SA+	SA−
SEM Δ ≥ 0.6	42	261	13	61	29	200
SEMΔ < 0.6	6	128	3	41	3	87
Sensitivity	87.5% (74.8%‐95.3%)		81.3% (54.4%‐96.0%)		90.6% (75.0%‐98.0%)	
Specificity	32.9% (28.3%‐37.8%)		40.2% (30.6%‐50.4%)		30.3% (25.1%‐36.0%)	
Bootstrap sensitivity (all locations) 87.4% (95% CI: 77.8%–96.7%)						
Bootstrap specificity (all locations) 33% (95% CI: 27.6%–38.7%)						
ROCAUC 0.6713 (CI: 0.5969, 0.7457); *P* < .0001						
Positive Predictive value (PPV) 14% (42/42 + 261)						
Negative predictive value (NPV) 96% (128/6 + 128)						
Per‐protocol analysis (n = 170)						
	All locations		Sacrum		Heel	
	SA+	SA−	SA+	SA−	SA+	SA−
SEM Δ ≥ 0.6	32	161	8	28	24	133
SEMΔ<0.6	6	79	3	20	3	59
Sensitivity	84.2% (68.8%‐94.0%)		72.7% (39.0%‐94.0%)		88.9% (70.8%‐97.7%)	
Specificity	32.9% (27.0%‐39.3%)		41.7% (27.6%‐56.8%)		30.7% (24.3%‐37.8%)	

*Note*: Matrix of true and false positive and negative diagnoses of pressure injury by the device (SEM) and the reference standard, clinical STA. A true positive is SA+/SEM+. A true negative is SA−/SEM−. A false positive is SA−/SEM+. A false negative is SA+/SEM−. Sensitivity and specificity are expressed as percentages with 95% confidence intervals; SA Δ ≥ 0.6 designates a positive diagnosis of pressure injury by skin and tissue assessment; SA Δ < 0.6 designates a negative diagnosis of pressure injury by skin and tissue assessment; SEM Δ ≥ 0.6 designates a positive diagnosis of pressure injury by the device; SEM Δ < 0.6 designates a negative diagnosis of pressure injury by the device.

Abbreviations: SEM, subepidermal moisture; STA, skin and tissue assessment; ITT, intent‐to‐treat.

### 3.4 Safety

The measure of analysis was the percentage of device‐related adverse events reported in the study. Adverse events in five enrolled subjects (2.6%) were reported. Four of the five were categorized by the principal investigators as unrelated to the study, with the remaining one being because of mortality from an underlying disease, also unrelated to the study. Of the 189 patients enrolled in the study, zero (0%) reports of adverse events were related to the use of the device or from prevention or treatments of PIs.

### 3.5 Early detection

Early detection of PI by device measurements was analyzed using the true positive subset of PI results (ie, PI diagnosed by ΔSEM Δ ≥0.6 and by STA). For 42 true positive PIs in the ITT population, ΔSEM Δ ≥0.6 occurred 4.7 days ±2.4 days earlier than diagnosis by STAs (Figure S5). For the sacrum, left heel, and right heel, detection by the device preceded diagnosis by STA by 4.7 ± 2.6, 5.1 ± 2.3, and 4.3 ± 2.4 days, respectively.

### 3.6 Characteristics of non‐PI skin changes

Visible or palpable changes in skin characteristics identified during STAs were documented for all participants (Table 6). Some changes indicative of PI (eg, redness or warmth) were documented but were not associated with a diagnosis of PI.

**Table 6:** Skin changes documented in the absence of diagnosed pressure injury

NPUAP/EPUAP/PPIA (2014, 1st ed.)	All	Sacrum	Heels
Red and nonblanchable skin	9	3	6
Red and blanchable skin	69	24	45
Other signs of skin changes (no PI)			
Changes in skin temperature	58	14	44
Changes in skin firmness	13	3	10

*Note*: Frequency of skin changes observed in cases in which pressure injury was ruled out during clinical skin and tissue assessment. Assessments were performed by wound care specialists with accreditation in wound and ostomy or tissue viability care.

Abbreviations: PI, pressure injuries.

## 4 DISCUSSION

In this longitudinal, blinded clinical study, measurements of the SEM biomarker using the device demonstrated sensitivity of 87.5% in identifying PIs, relative to the reference standard of STA by wound care specialists. Additionally, the device produced a positive finding 4.74 days ±2.39 days earlier than the diagnosis of a PI by STA (Figure S5). These data agree with the temporal delay of 3‐10 days between SEM changes and the appearance of visible or palpable skin changes demonstrated in other studies.^16^, ^17^, ^18^, ^19^, ^20^, ^21^

This study did not meet the targeted endpoint for specificity of at least 55%. Two hundred and sixty‐one (n = 261) positive SEM deltas were classed as false positives resulting in a specificity approaching 33%. Given the comparison of nonvisual to visual skin damage, it is not surprising that a lower specificity is observed in this study. The Investigators considered a range of explanations for these specificity results.

1. Clinical judgment as an inadequate index value: Risk assessment tools have good predictive capacity, moderate sensitivity and specificity but have highly variable inter‐rater reliability.^12^, ^13^, ^47^ Clinical judgment has moderate sensitivity and specificity but has poor predictive capacity and inter‐rater reliability.^48^ This study utilized clinicians with a high level of experience in STA (to represent as the “gold standard” comparator); nonetheless, such assessments are subjective and not the error‐free requirement of an index value necessary for complete evaluation of a new diagnostic test. A true assessment of the biomarker is classed as a negative PI result in this test if the PI did not manifest.
2. Reversibility and self‐resolution before the damage threshold is reached: Research by Oomens et al showed, “…tissue damage is initiated at a cellular level” and, “unloading the tissue will restore the supply of oxygen and nutrients to the tissue” to return tissue to homeostasis.^33^ Further, Halfens^31^ noted subgroups of patients with grade 1 pressure ulcer when they were evaluated over multiple days: 22.1% resolved, 22.1% deteriorated, 35.3% unchanged, and 20.6% disappeared (thought to be an initial misdiagnosis or resolution). Although it is not possible to distinguish between damage that will and will not reverse, application of the right interventions provided before the damage threshold is reached results in tissue “resetting” to “normal homeostasis,” namely the restoration of oxygen supply and nutrients to the tissue and removal of waste products.^31^, ^33^ In addition, Swisher et al^32^ published a rat study demonstrating that impedance technology could measure nonvisible tissue damage also showed that pressure damage to tissue can and will reverse when the tissue is unloaded early enough in the damage cascade.
3. Potentially Confounding effects of Interventions: In this study, it would have been unethical to withhold prevention measures. Preventive care can significantly reduce the incidence of pressure ulcers.^49^, ^50^ All (100%) of subjects received some form of preventative interventions. The high level of offloading measures noted in the study potentially led to reversals of tissue damage. Intensive forms of offloading measures (repositioning every 1 or 2 hours, heel boots and elevations and active and low air mattress support systems) were provided to 89.6% of the enrolled subjects while 10.4% received less intense forms of preventive care (eg, static bed mattress, topical agents, less turning frequency). The conundrum for test validation trials where preventative measures are ethically necessary has been previously discussed by DeFloor and Grypdnock in their critique of the validity of using risk assessment scales, “If preventative measures are used, the probability that a patient will develop a pressure ulcer at the start of the study will not remain constant until its end.” Further, “If some patients at risk (according to the scale) and some patients not at risk (according to the scale) receive preventive measures, pressure ulcer incidence will probably decrease in both groups, but not necessarily to the same extent”.^51^, ^52^ Finally, “The number of true positives will decrease and the number of false positives will increase.”
4. Delta performance: In determining the optimal cutoff to use for clinical interpretation of the SEM delta, a range of cutoffs was considered. The SEM delta of Δ ≥0.6 cutoff was chosen to prioritize sensitivity over specificity; a developed PI being potentially catastrophic, while preventative interventions presenting negligible risk and costs. A review of the computation of SEM delta cutoffs using data from this study is summarized in Table 7. Different delta cut‐off values change the computed sensitivities and specificities.
5. Potentially missed diagnoses of PIs: Four percent (4%; n = 7/182) of the ITT population were not classed as PIs, however, when the subconditions of STA described in the international guidelines are applied these would have been classified as a pressure injury (Tables 5 and 6). Classifying these assessments as PIs, results in sensitivity of 89.09% (95% CI: 77.75%‐95‐89%), and specificity of 33.51% (28.79%‐38.49%).

**Table 7:** Range of SEM Delta

SEM Δ	ITT (N = 182)					
	Sensitivity			Specificity		
	n	%	95% CI	n	%	95% CI
Δ ≥0.6	42	87.5	74.8%, 95.3%	124	32.6	27.9%, 37.5%
Δ ≥0.7	39	81.3	67.4%, 91.1%	170	44.6	39.6%, 49.8%
Δ ≥0.8	32	66.7	51.6%, 79.6%	227	59.6	54.5%, 65.6%

Abbreviations: SEM, subepidermal moisture; ITT, intent‐to‐treat.

Results from this study illustrate the difficulties inherent to diagnosis of PI by visible or palpable skin changes.^53^, ^54^ In the present study, controls were implemented to improve inter‐rater reliability, including the use of wound care specialists with accreditation in wound and ostomy or tissue viability care to perform protocol‐driven STAs. Nevertheless, a number of skin changes indicative of PI, particularly red and blanchable lesions, warmth/coolness, and firmness, were noted consistent with the second Edition of the 2014 international guidelines^11^ but not diagnosed as PI (Table 6). The second edition and repeated in the 2019 international guidelines^43^ suggests assessment of blanchable erythema and changes in sensation, temperature, or firmness may precede visual changes associated with a later stage 1 PI.

Subjects in this study were at PI risk and all were receiving universal prevention interventions, and in some instances, anatomy‐specific interventions. Blinding assured that no additional interventions were prescribed for patients as a result of positive SEM deltas. The clinical‐practice implications of the SEM false‐positive rate, while beyond the scope of this clinical trial, are important to consider. A positive SEM delta is an indicator that a patient's skin and tissue at the particular anatomy is exhibiting the signs of incipient damage which may progress toward a PI. For patients who have elevated SEM deltas, interventions may include more frequent anatomy‐specific offloading, a heel boot, 30° wedge, or prophalactic dressing.

False negative rates were low (n = 6; 3.3%). The risk of a false negative outcome is that a patient continues to receive the current standard of care. The likelihood of a false negative outcome is low based on this study results; patients will continue receiving standard of care interventions.

**Figure 1:**
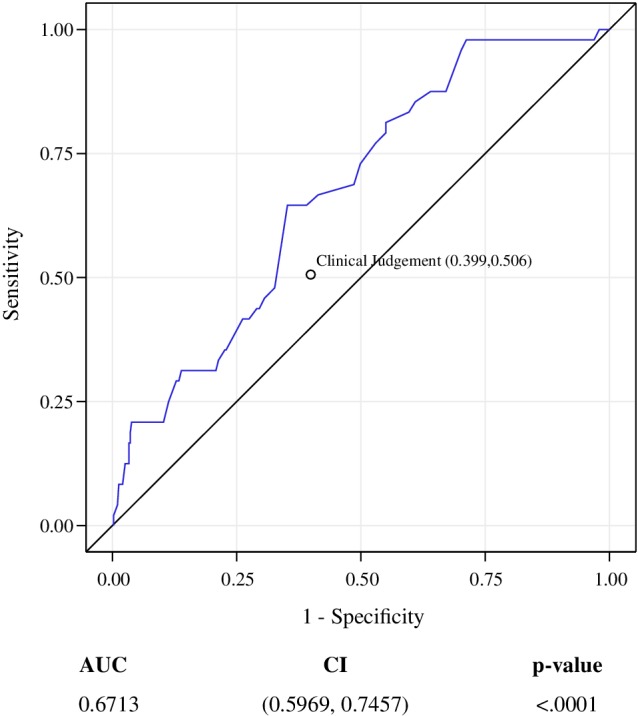
Receiver operating characteristic curve for performance of the study device relative to the gold standard of skin and tissue assessment. Receiver operating characteristic curve illustrating diagnostic sensitivity and specificity of the investigational device in detecting pressure injury. AUC, area under the curve. CI, confidence

The AUC statistic of 67% shows a combined sensitivity and specificity of the test of SEM as one demonstrating clinical utility exceeding that of clinical judgment alone. Clinical judgment, as a unique diagnostic method, and as a collective term for STA combined with nurse interpretation, when plotted on the ROC curve is shown to the lower right side of the ROC curve for SEM data (Figure 1). Unique true‐positive, true‐negative, false‐positive, false‐negative results were not available for clinical judgment, hence the single plot of 1‐specificity (1‐60.1%) and sensitivity (50.6%).^13^

The PPV of 14% is the proportion of true positive SEM deltas at heels and sacrum from the first SEM positive observation which later manifested into a confirmed PI. All SEM positive values preceded or coincided with PI diagnosis by STA. The same discussion points applicable to understanding the specificity percentage apply to the understanding the PPV percentage. Precision of the SEM test was potentially confounded by the preventative interventions necessarily applied to all patients. The negative predictive value of 96% is the proportion of true negative SEM deltas at heels and sacrum, which did not later manifest into confirmed PI. The 96% result suggests a higher certainty that normal SEM deltas (0.5 or less) in combination with prescribed preventative interventions will not result in a PI over the assessed anatomy, although such a result is not always true.

Future studies should assess alternative cut‐off delta values, particularly for anatomies with skin and tissue histology different to those found at heels and sacrum. Future interventional studies should explore the preventative effect of interventions precipitated by clinical judgment informed by SEM deltas. Formal Health Econonomic analyses should be undertaken to evaluate the cost consequences of the false positive rate, in particular.

## 5 CONCLUSIONS

Latency between the initial onset of pressure damage and the subsequent manifestation of a visible and clinically significant injury at the skin's surface, and the application of anatomy‐specific interventions has challenged clinical practice, and the objective of PI prevention for decades.^55^ Of all reported Hospital Acquired Conditions, PIs are the only conditions to have worsened in the United States^6^ between 2012 and 2017, and they remain the most reported type of patient harm in the UK's National Health Service.^56^ Keeping patient's tissue health is of critical clinical importance but remains a pernicious clinical challenge in patients who are at risk for PIs.

Sensitivity of the device exceeds that of visual STAs alone in its ability to detect the antecedents of a developing PI at the particular scanned anatomies. These results corroborate findings in other SEM studies.^20^ Sensitivity and specificity of the SEM test in the aggregate as measured by the 67.13% area under the curve exceed that of clinical judgment alone. Even acknowledging specificity limitations, these data suggest that SEM biocapacitance measures can complement visual STAs, facilitate earlier identification of the risk of specific anatomies developing PIs, and inform earlier anatomy‐specific intervention decisions than visual STAs alone.

The use of specificity as an end point was recognized, before study inception, as a worst‐case assessment for the SEM test because it classes all results in which a PI did not visibly manifest (STA negative) but where changes in SEM were observed (SEM positive) as false positive results. Given proper ethical requirements of uninterrupted PI prevention protocols, which may have confounded specificity results in this study, future like studies require a different epistemological method to completely assess the specificity of SEM. An objective gold‐standard reference index test is required to perform such a test. Until such time and recognizing the false positive rate, these data suggest the clinical role of the device is in informing practitioners of anatomy‐specific PI risk where clinical judgment retains primacy over diagnosis.

Even though not all anatomies exhibiting elevated SEM deltas will proceed to eventually develop a PI, it is important for health‐care providers to be aware of the early warning signs so they can take risk‐appropriate mitigating steps. PI often occur without prior visual and palpable cues appearing in time to prevent them, especially if the injury is not superficial. Studies evaluating whether commencing PI prevention activities (eg, anatomy‐specific offloading) on the basis of positive SEM deltas as an adjunct to clinical judgment, rather than solely on risk and visual STAs have shown material reductions in the incidence of PIs.^26^ As noted earlier, further analyses on prevention bundles and cost‐consequences are still necessary in future work.

## CONFLICT OF INTEREST

Henry Okonkwo, Dr. Ruth Bryant and Jeanette Milne presented at Bruin Biometrics' sponsored symposia, for which they were paid speakers fees and reimbursed expenses by the Company.

**Abbreviations**
PI: pressure injury
SEM: subepidermal moisture
VSA: visual skin and tissue assessment
ITT: intent‐to‐treat
PP: per protocol
eCRF: electronic Case Report Form
EDC: electronic data capture
USA: United States of America
UK: United Kingdom
IAD: incontinence associated dermatitis

## Supporting information


**Supplementary Figure 1** Sacrum SEM Scanner 200 Read Locations

**Supplementary Figure 2** Heel SEM Scanner 200 Read Locations

**Supplementary Figure 3** SEM Scanner 200

**Supplementary Figure 4** Study Participant Flow Diagram

**Supplementary Figure 5** Pressure ulcers are diagnosed earlier with SEM Scanning than with skin assessments alone

## Endnote

*
This sentence reproduced by permission of the authors.
