# Supplementary figures and images for: A blinded clinical study using a subepidermal moisture biocapacitance measurement device for early detection of pressure injuries
Source: Wound Repair Regen. 2020 Jan 21;28(3):364–74. doi: 10.1111/wrr.12790 (PMC7217158; doi:10.1111/wrr.12790)

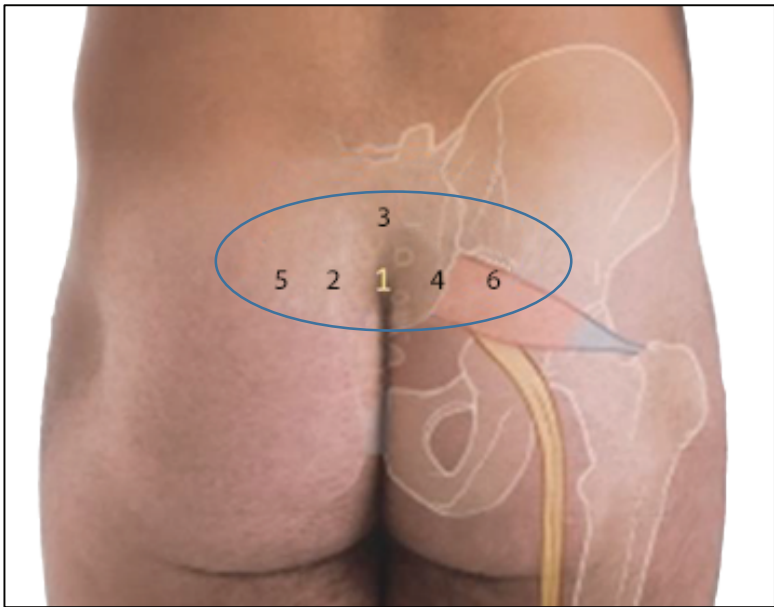

Supplement: Supplementary file 1 — Supplementary Figure 1 Sacrum SEM Scanner 200 Read Locations [file WRR-28-364-s001.pdf]

**A**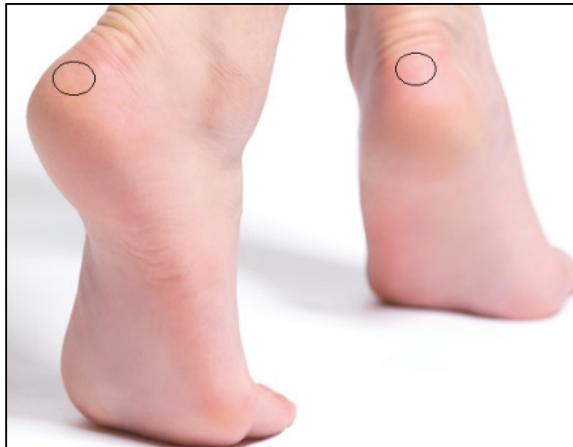**B**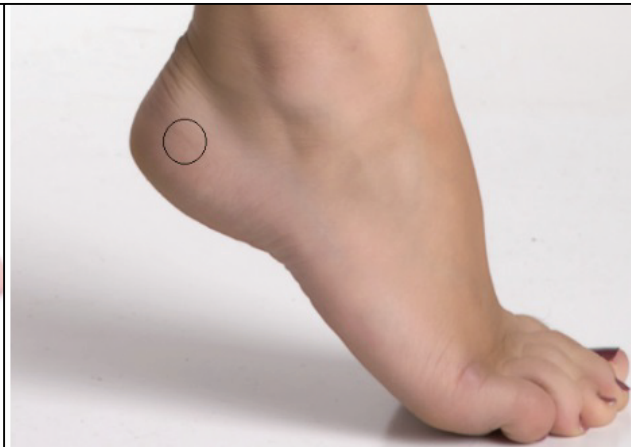**C**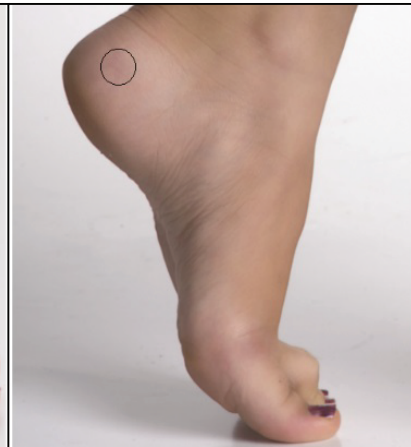**D**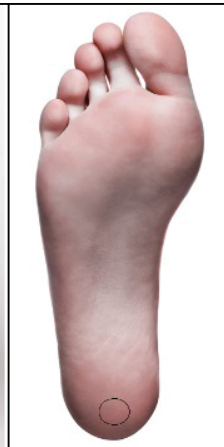

Supplement: Supplementary file 2 — Supplementary Figure 2 Heel SEM Scanner 200 Read Locations [file WRR-28-364-s002.pdf]

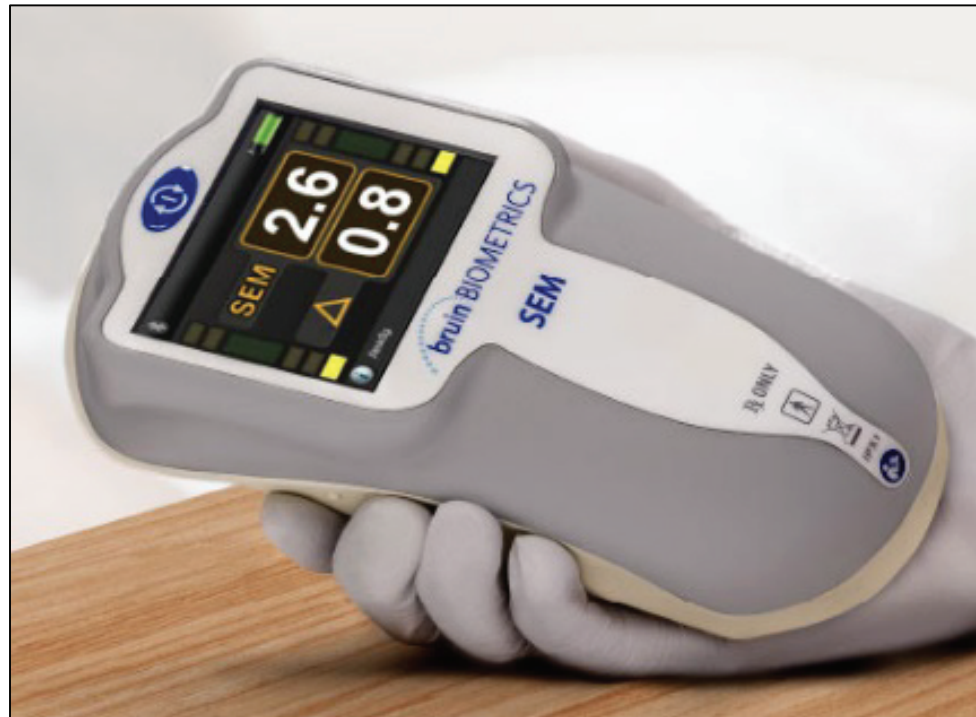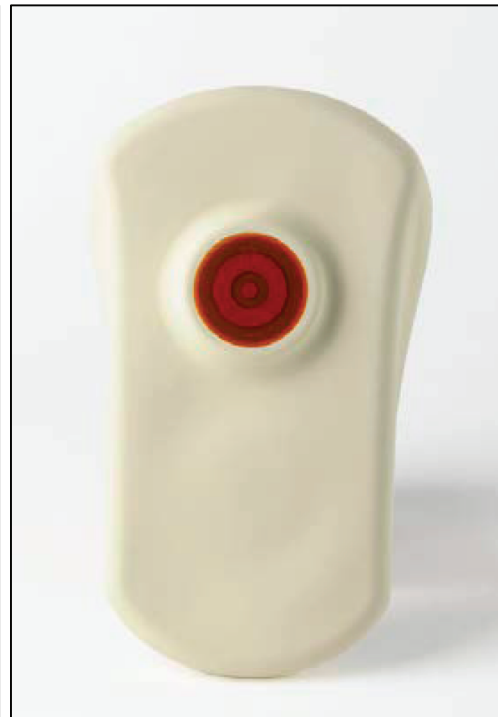

Supplement: Supplementary file 3 — Supplementary Figure 3 SEM Scanner 200 [file WRR-28-364-s003.pdf]

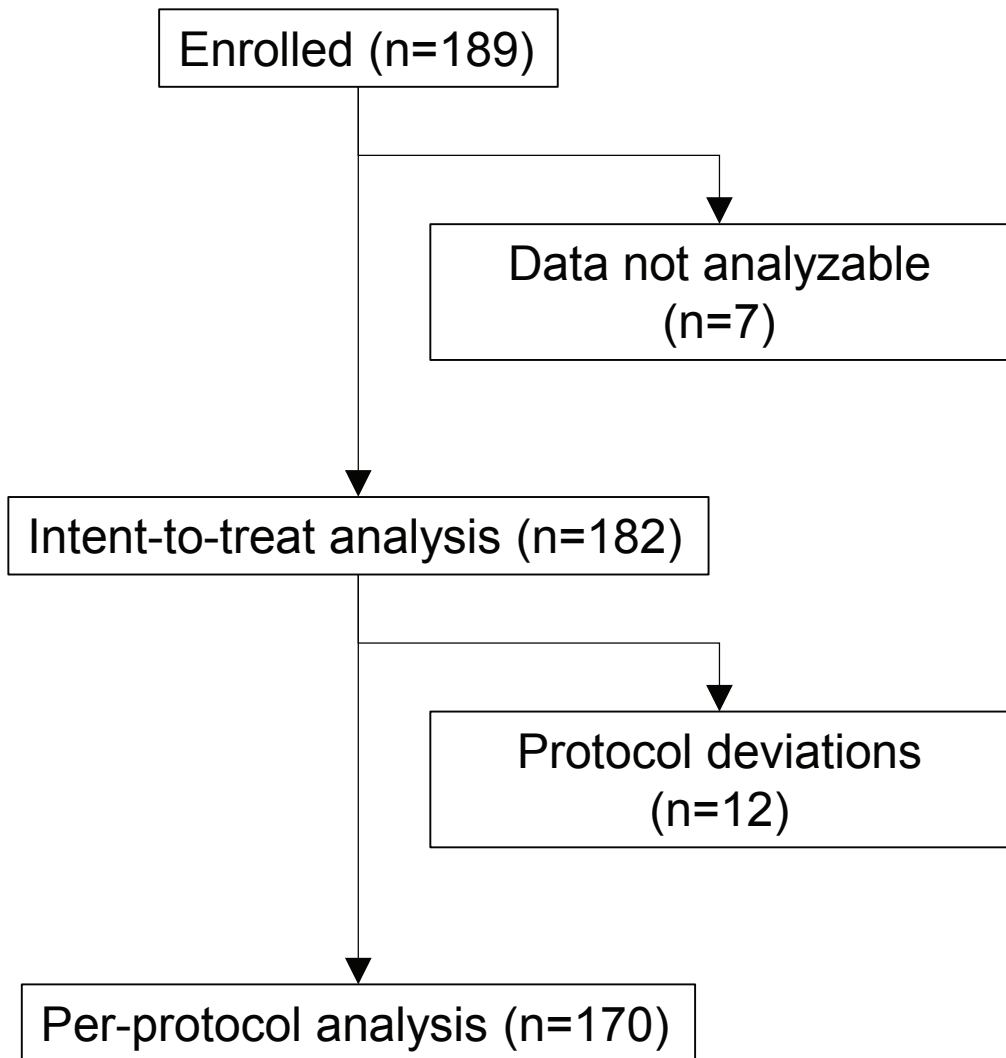

Supplement: Supplementary file 4 — Supplementary Figure 4 Study Participant Flow Diagram [file WRR-28-364-s004.pdf]

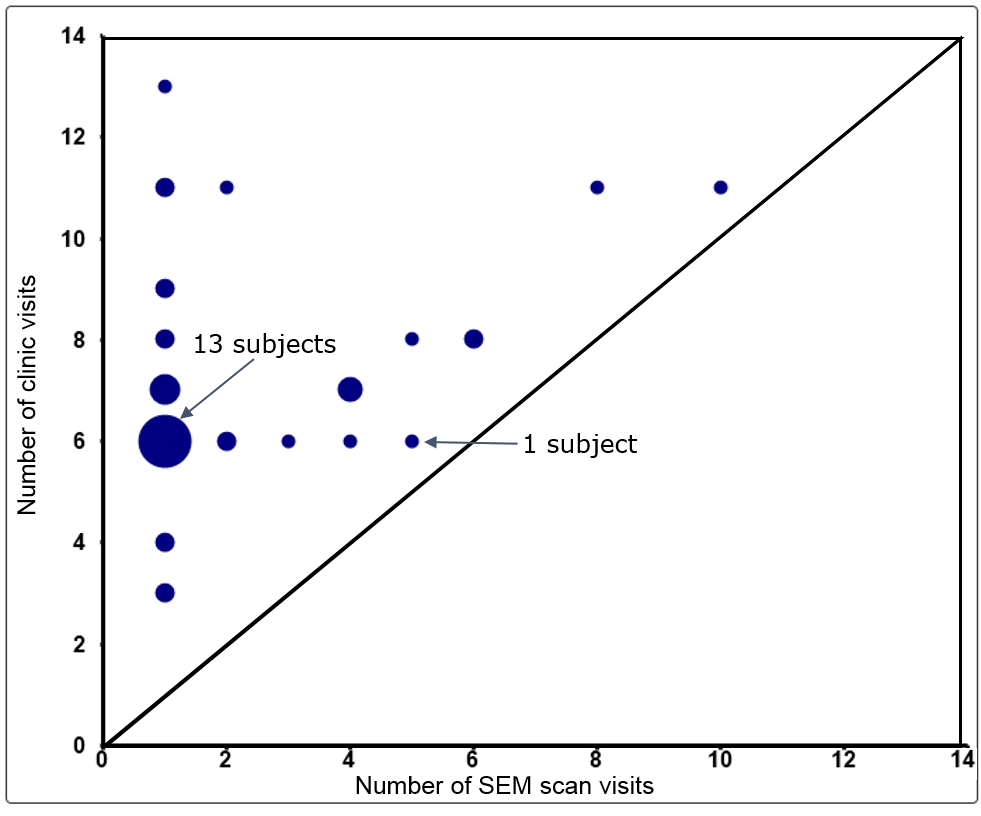

Supplement: Supplementary file 5 — Supplementary Figure 5 Pressure ulcers are diagnosed earlier with SEM Scanning than with skin assessments alone [file WRR-28-364-s005.tif]
